# Chloroplast development and genomes uncoupled signaling are independent of the RNA-directed DNA methylation pathway

**DOI:** 10.1038/s41598-020-71907-w

**Published:** 2020-09-22

**Authors:** Liangsheng Wang, Dario Leister, Tatjana Kleine

**Affiliations:** Plant Molecular Biology (Botany), Department Biology I, Ludwig-Maximilians-Universität München, Planegg-Martinsried, Germany

**Keywords:** Light responses, Plant development

## Abstract

The Arabidopsis genome is methylated in CG and non-CG (CHG, and CHH in which H stands for A, T, or C) sequence contexts. DNA methylation has been suggested to be critical for seed development, and CHH methylation patterns change during stratification and germination. In plants, CHH methylation occurs mainly through the RNA-directed DNA methylation (RdDM) pathway. To test for an involvement of the RdDM pathway in chloroplast development, we analyzed seedling greening and the maximum quantum yield of photosystem II (F_v_/F_m_) in *Arabidopsis thaliana* seedlings perturbed in components of that pathway. Neither seedling greening nor F_v_/F_m_ in seedlings and adult plants were affected in this comprehensive set of mutants, indicating that alterations in the RdDM pathway do not affect chloroplast development. Application of inhibitors like lincomycin or norflurazon inhibits greening of seedlings and represses the expression of photosynthesis-related genes including *LIGHT HARVESTING CHLOROPHYLL A/B BINDING PROTEIN1.2* (*LHCB1.2*) in the nucleus. Our results indicate that the *LHCB1.2* promoter is poorly methylated under both control conditions and after inhibitor treatment. Therefore no correlation between *LHCB1.2* mRNA transcription and methylation changes of the *LHCB1.2* promoter could be established. Moreover, we conclude that perturbations in the RdDM pathway do not interfere with *gun* signaling.

## Introduction

Chloroplasts are the hallmark of algae and plants. In addition to their most prominent role as organelles of photosynthesis, they host numerous metabolic reactions involving amino acids, lipids, sulfur, isoprenoids, vitamins, as well as the production of some hormones or their precursors^1^. They are therefore essential for plant growth and development. In the course of their evolution as intracellular organelles, plastids have lost most of their genes to the nucleus. They have retained a genome comprising only around 100 genes^2^, but contain at least 1,800 nucleus-encoded proteins^3^. Plastids are therefore semi-autonomous organelles, as they are dependent on products synthesized in the nucleocytoplasmic compartment for the assembly of multiprotein complexes of mixed genetic origin, such as the gene expression machinery or the photosynthetic apparatus. Plastid biogenesis and maintenance of chloroplast functions are highly complex processes that are not yet fully understood. Clearly, stringent coordination of the expression of the plastid and nuclear genomes is required, which is achieved by bidirectional information exchange.

Because most plastid proteins are encoded in the nucleus, the bulk of the information flow during chloroplast biogenesis is from the nucleus to the organelle^4^. The nucleus thus exercises anterograde control over the plastids. Conversely, during retrograde signaling, plastids are the sources of specific signaling components that transmit information to the nucleus, which modulates nuclear gene expression (NGE) in accordance with the developmental and physiological states of plastids^5–7^. Retrograde signals can be classified as 'biogenic control' signals which are active during early stages of chloroplast development, while 'operational control' functions in response to environmental fluctuations^8^. Previous analyses that sought to understand how retrograde signals control NGE have focused almost exclusively on transcriptional regulation. However, recent evidence indicates that changes in NGE due to operational retrograde signaling are not only a consequence of differential transcript accumulation, but can involve alternative splicing^9^, read-through events^10^, and microRNA biogenesis^11^. With the ongoing refinement of Next Generation Sequencing technologies, we can expect the discovery of further interesting findings in this context. As an example, RNA-seq, in combination with DNase I hypersensitive site sequencing, has revealed that the level of chromatin condensation during extended periods of darkness is correlated with the repression of light- and photosynthesis-related genes. Those affected by changes in chromatin condensation are enriched in genes related to plastid signaling and their promoters frequently contain GOLDEN2-LIKE1 (GLK1) binding elements^12^. GLK1 and GLK2 transcription factors coordinate the expression of the photosynthetic apparatus^13^. Since DNA methylation is a DNA modification that can affect gene expression, another interesting possibility is that alterations in DNA methylation are involved in modulating NGE in response to chloroplast-derived signals. Alternatively, the perception of light, or lack thereof, could induce changes in DNA methylation of nuclear genes associated with chloroplast biogenesis, or their regulators, thus influencing chloroplast development^14^.

The expression of plastid-encoded genes is not sensitive to DNA methylation^15^, but the nuclear genome of Arabidopsis is methylated in both CG and non-CG (CHG, and CHH in which H stands for A, T, or C) sequence contexts, and the global DNA methylation state is defined by de novo methylation, maintenance of methylation and active demethylation^16^. De novo DNA methylation and maintenance thereof in plants is mediated by the RNA-directed DNA methylation (RdDM) pathway^17^ and DNA methylases some of which have been named DDM before, in reference to a decrease in DNA methylation in *ddm* mutants^18^. According to the current view, the canonical RdDM pathway is initiated by RNA polymerase IV (POL IV)-mediated transcription of non-coding RNAs, which is followed by RNA-DEPENDENT RNA POLYMERASE 2 (RDR2)-dependent copying of the transcript to generate a double-stranded RNA (dsRNA). DICER-LIKE (DCL) proteins, mainly DCL3, then cleave these dsRNAs into siRNAs. The siRNAs are loaded onto ARGONAUTE (AGO) proteins, mainly AGO4 and AGO6, and pair with complementary scaffold RNAs, which are transcripts produced by POL V. AGO4 interacts with the DNA methyltransferase DOMAINS REARRANGED METHYLASE 2 (DRM2), which catalyzes de novo DNA methylation in a sequence-independent manner^16^. Following DNA replication, CG, CHG and CHH methylation are maintained by METHYLTRANSFERASE 1 (MET1), CHROMOMETHYLASE 3 (CMT3), and CMT2 respectively^19^. Maintenance of CHH methylation through RdDM is mediated through DOMAINS REARRANGED METHYLTRANSFERASES 1 (DRM1) and 2 (DRM2). KRYPTONITE (KYP/SUVH4), SUVH5, and SUVH6 are the primary histone H3K9 methyltransferases and are required for CMT3 and CMT2 function^16,17,19,20^. DNA methylation can be reversed by active demethylation, which is initiated by DNA demethylases, of which REPRESSOR OF SILENCING 1 (ROS1) is the major representative in *Arabidopsis thaliana*^21^.

Despite the critical significance of chloroplast biogenesis for plant development and performance, our understanding of how functional chloroplasts are initially established remains incomplete, and new layers of control are still being considered. This holds also and especially true for the biogenic type of *genomes uncoupled* (*gun*) signaling. This type of signaling is known for three decades^22^, but its mechanism is still elusive and corresponding experimental results and their interpretation are disputed^23^. One such proposal concerns the potential relationship between DNA methylation changes, chloroplast development and the expression of nucleus-encoded photosynthesis genes. Here, we have employed mutants defective in components of the canonical RdDM pathway to ascertain whether they contribute to the regulation of chloroplast biogenesis and/or biogenic signaling. The results described below show that RdDM-mediated DNA methylation changes have no influence on chloroplast development or the expression of nuclear marker genes of retrograde signaling.

## Results

### Messenger RNA expression levels of genes involved in methylation processes during chloroplast development

**Figure 1:**
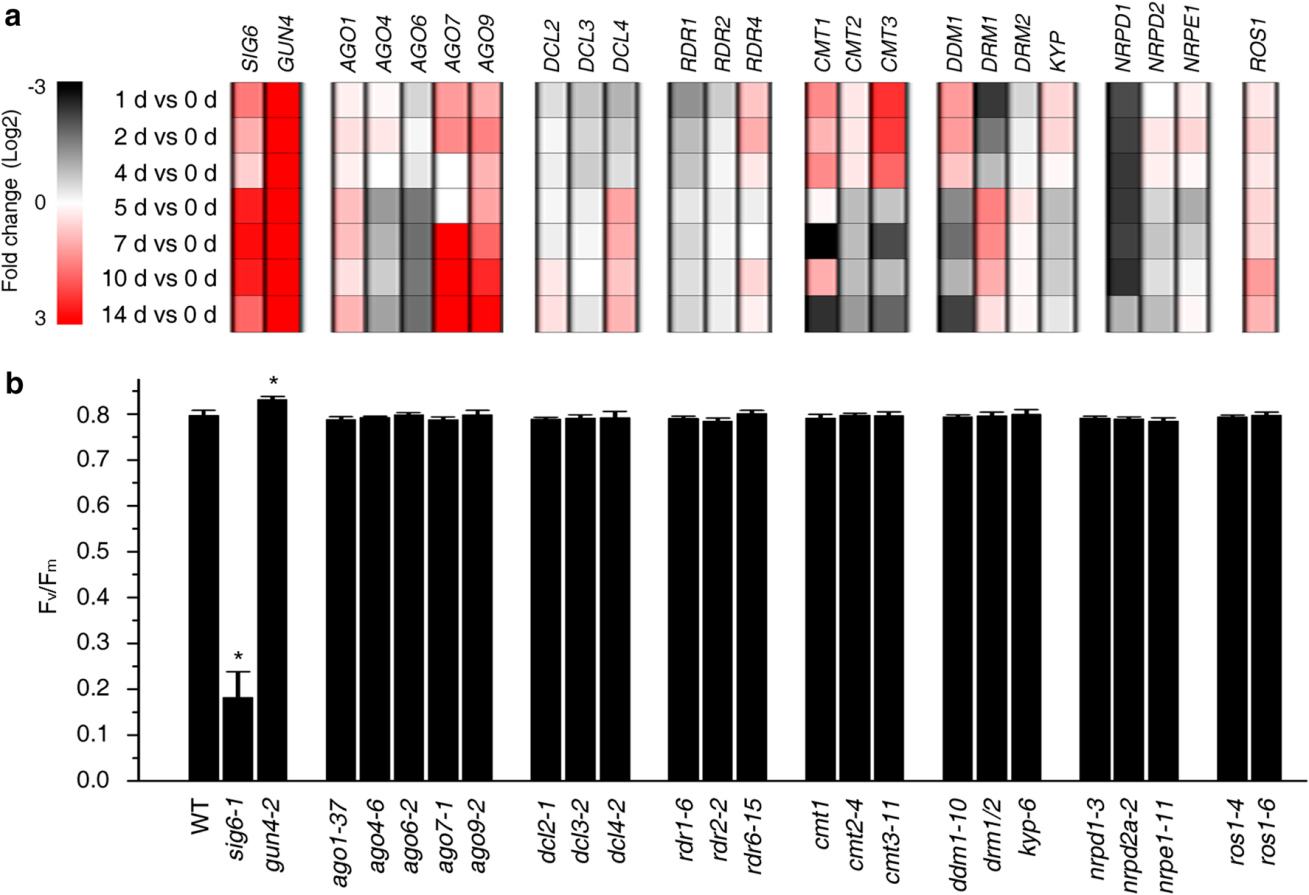
Messenger RNA levels and photosynthetic behavior of mutants for components of (de)methylation pathways. **(a)** Heatmap showing relative changes in the mRNA expression of genes involved in the RdDM pathway and of genes selected because of their mRNA expression profile in an Arabidopsis cell culture after the onset of light in a time series of 1 to 14 days^28^. Log_2_-fold changes are calculated relative to gene expression values at day 0. **(b)** The maximum quantum yield of PSII (F_v_/F_m_) was measured with an imaging Chl fluorometer (Imaging PAM). Seedlings were grown on half-strength MS medium for 4 days under LD conditions, and were dark-adapted for 10 min before transient chlorophyll fluorescence was measured. The *sig6-1* and *gun4-2* mutants were used as controls. Data represent mean values from at least 20 seedlings. Significant differences (*t*-test*; p* < 0.05) with respect to the WT are indicated by asterisks.

During embryogenesis of many oilseed crops including *Arabidopsis thaliana* (Arabidopsis), photosynthetically active chloroplasts are detected in embryos at the globular, heart, torpedo and walking-stick stages^24^, and are most abundant at 6–12 days after fertilization^25^. In a subsequent desiccation phase leading to the production of dry seeds, these chloroplasts dedifferentiate, and chloroplasts again develop after germination^26^. If RdDM plays a role in chloroplast development, this might be reflected in changes in the transcriptional activity of genes encoding proteins involved in the RdDM pathway (see “Introduction”). To test this, we analyzed previously published RNA-Seq data from dry seeds and seeds/seedlings at 0, 1, and 2 days after imbibition (DAI)^27^ and from an Arabidopsis cell culture after the onset of light for a time series of 1 to 14 days^28^. As a control, mRNA expression patterns of *GENOMES UNCOUPLED4* (*GUN4*) and *SIGMA FACTOR 6* (*SIG6*) were also followed. GUN4 stimulates chlorophyll biosynthesis^29^, while SIG6 is associated with the plastid-encoded prokaryotic-like RNA polymerase complex and promotes early chloroplast development in cotyledons^30^. Moreover, we included genes for proteins involved in other epigenetic processes like histone (de)methylation, chromatin remodeling, and small RNA biogenesis^31^. In dry seeds, mRNA expression levels were generally low with the exception of a few genes, including *DRM1*, whose protein product is responsible for the maintenance of CHH methylation (see Supplementary Table S1 online). After seed imbibition, the levels of *GUN4* and *SIG6* mRNAs, and transcripts of most of the genes for epigenetic factors, increased to varying extents. This increment was observed for genes belonging to the various epigenetic pathways and was not restricted to one process. Focusing on light-induced expression changes in the cell culture, a two-phase pattern can be discerned for some of the genes (see Supplementary Fig. S1). This pattern was previously recognized as a pronounced difference in expression behavior between days 4 and 5^28^. For example, *DECREASED DNA METHYLATION 1* (*DDM1*), *CMT2*, *CMT3* and *NUCLEAR RNA POLYMERASE D2* (*NRPD2*) were up-regulated on days 1, 2 and 4, but down-regulated from day 5 on (Fig. 1a, see Supplementary Fig. S1, Supplementary Table S1 online). Other genes were constantly up-regulated (*AGO1*, *AGO9* and *ROS1*) or down-regulated (*AGO3*, *AGO6*, *RDR1*, *NRPD1*, *NRDP2B*) after the onset of light. *GUN4* and *SIG6* showed strong and moderate up-regulation of their mRNA levels respectively over the whole time-course. Overall, this analysis revealed that several genes involved in epigenetic processes are differentially expressed throughout chloroplast development.

### Chloroplast development is not disturbed in mutants of the RdDM pathway

During embryogenesis, extensive CHH DNA methylation occurs^27,32^, which is maintained in adult plants^32^. But no significant changes in methylation in CG and CHG contexts were observed at any stage of embryo development, suggesting that especially CMT2 and the RdDM pathway might be active during embryogenesis (see “Introduction”). Therefore, to test whether altered RdDM-mediated methylation patterns have an impact on chloroplast biogenesis, we decided to investigate mutants in the classical RdDM pathway more closely. In addition, because the expression behavior of *AGO7* and *AGO9* clustered with that of *SIG6* (see Supplementary Fig. S1), amounts of *AGO7*, *AGO9* and *ROS1* mRNAs were continuously upregulated during the whole time course studied by Dubreuil et al.^28^, and *CMT1* and *CMT3* were markedly upregulated throughout the first phase (days 1 to 4) (see Supplementary Fig. S1, Supplementary Table S1 online), mutants thereof were also included. With this selection of mutants, we also consider those that are involved in methylation maintenance during DNA replication. It is very likely that cell divisions and therefore DNA replication occur in a cell-culture based system used by Dubreuil et al.^28^. But it has to be noted here that although Arabidopsis cotyledons expand considerably and perform photosynthesis after light exposure, it is thought that the number of cells in Arabidopsis cotyledons is already determined during the embryonic phase^33^. However, this claim is highly disputed (discussed in^34^). By systematically studying cell division in Arabidopsis cotyledons, it was found that palisade mesophyll cells of dark-grown seedlings started to divide between day 2 and 3, but in 5-day-old light-grown seedlings no cell division was observed^33^. Therefore and because of reasons stated along with the respective experiment, three different set-ups will be used in the following: 4-day-old light-grown seedlings, 4-week-old plants, and seedlings grown for 3 days in darkness and then transferred into continuous light. Chlorophyll autofluorescence was used as a marker for the appearance of chloroplasts during embryogenesis^24^, and F_v_/F_m_, the maximum quantum yield of photosystem II (PSII), can be used as a proxy for photosynthetic activity and photosystem biogenesis. Here, to investigate chloroplast development, F_v_/F_m_ was determined in 4-day-old seedlings with the selected mutant lines, along with the WT (Col-0), and *sig6-1* and *gun4-2* mutants that served as controls (Fig. 1b, see Supplementary Fig. S2). F_v_/F_m_ was significantly depressed to ~ 0.2 in *sig6-1* compared to a wild-type (WT) value of 0.8, confirming that chloroplast development is perturbed in this mutant. In *gun4-2*, F_v_/F_m_ was slightly higher than in WT, but F_v_/F_m_ values in the other mutants were not significantly different from the WT value (Fig. 1b). Chloroplast development in true leaves might differ from chloroplast development in cotyledons because, in true leaves, chloroplasts develop from meristematic proplastids when the leaf primordia emerge ^13,26^. However, measurement of F_v_/F_m_ in leaves of 4-week-old plants did not reveal any significant differences compared to the WT (see Supplementary Fig. S3).

**Figure 2:**
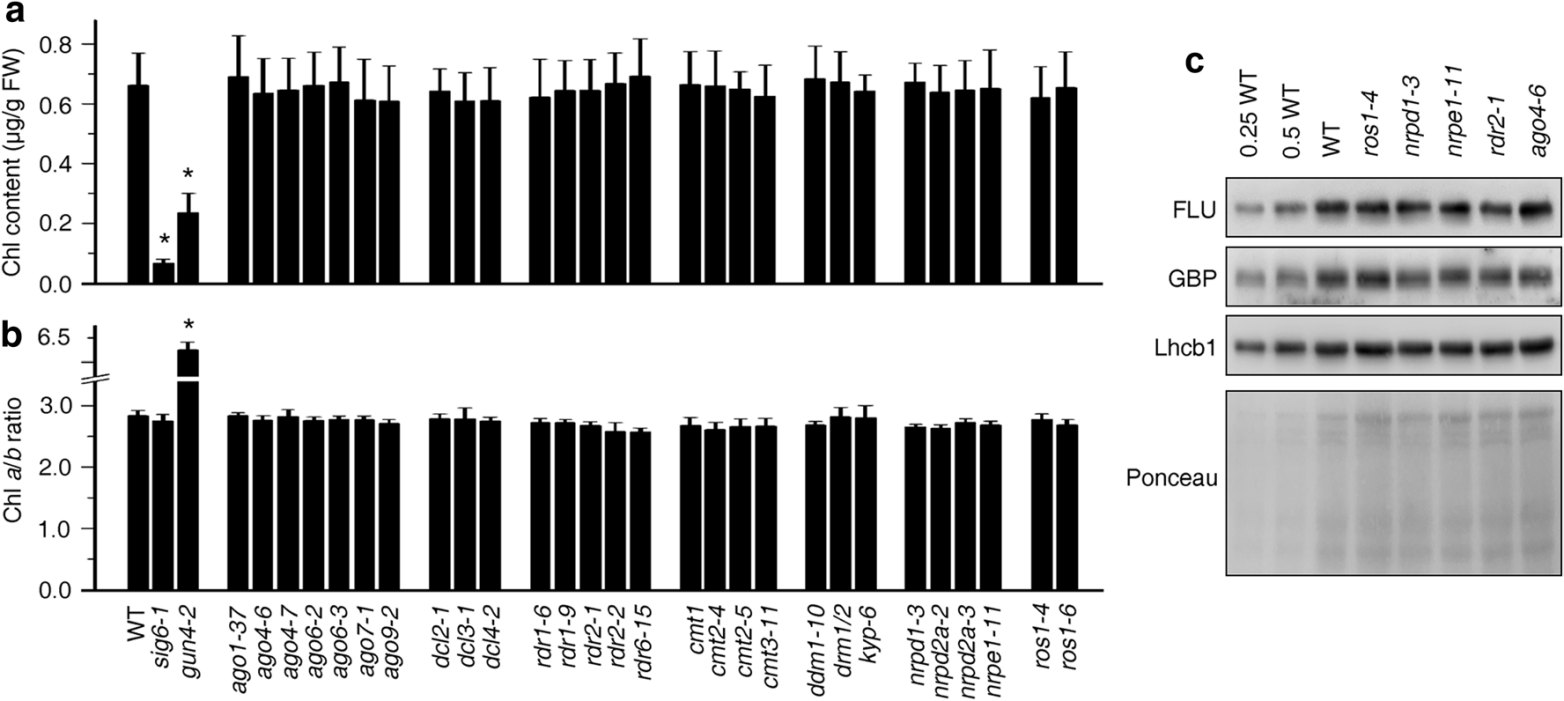
Perturbation of the RdDM pathway does not significantly affect chlorophyll biosynthesis. (**a**) Determination of total chlorophyll (Chl *a* + *b*) contents of 4-day-old seedlings grown under LD conditions. Chlorophyll was acetone-extracted and measured spectrophotometrically, and concentrations were determined as described (see “Methods”). Data are shown as mean values ± SD from 6 different plant pools. Each pool contained more than 100 seedlings. Significant differences (*t*-test; *p* < 0.05) with respect to Col-0 are indicated by asterisks. (**b**) Graph displaying the Chl *a*/*b* ratio of chlorophylls extracted in (**a**). **(c)** Immunoblot analysis of representative enzymes involved in chlorophyll biosynthesis (FLU and GBP) and the light-harvesting chlorophyll *a*/*b* binding protein Lhcb1. Total protein extracts from 4-day-old seedlings obtained from the wild-type (Col-0) and representative (de)methylation mutants were fractionated by SDS-PAGE, and blots were probed with antibodies raised against the individual proteins. Increasing levels of wild-type proteins were loaded in the lanes marked 0.25 WT, 0.5 WT and WT. The relative loading amounts of each sample were visualized by staining the blot with Ponceau S. FLU, FLUORESCENT IN BLUE LIGHT; GBP, glutamyl-tRNA reductase-binding protein. The figure was assembled from different blots (delineated by a black rectangle) and full-length blots are presented in Supplementary Fig. S6.

The regulation of chloroplast greening and photosystem biogenesis are not entirely coupled, but are controlled by separate systems^35^. Therefore, we tested whether a defect in the RdDM pathway might impinge on chloroplast greening by measuring Chl contents of 4-day-old seedlings. While the total Chl (Chl *a* + *b*) content was significantly reduced in *sig6-1* and *gun4-2* seedlings, no difference from the WT could be detected in the other mutant seedlings (Fig. 2a). This was also true of the Chl *a*/*b* ratio (Fig. 2b) which can be used—together with F_v_/F_m_—as a proxy for the general composition of the photosynthetic apparatus and chloroplast development. CHH methylation is virtually eliminated in nuclear RNA polymerase IV (*nrpd1*), and V (*nrpe1*) subunit mutants, and *rdr2* and *ago4* mutants^20^, and CHH sites are hypermethylated in *ros1* mutants^36^. For example, loss-of-function mutations in *ROS1* result in hypermethylation of the *DESICCATION-RESPONSIVE PROTEIN 29A* (*RD29A*) promoter and silencing of a *RD29A*-promoter-driven luciferase transgene^37^. To further investigate a possible effect of alterations of methylation patterns on chloroplast development, we focused in the following on these mutants. Immunoblot analysis was performed on total protein extracts of 4-day-old Col-0 and *ros1-4*, *nrpd1-3*, *nrpe1-11*, *rdr2-1*, and *ago4-6* mutant seedlings to test whether levels of proteins involved in chlorophyll biosynthesis or that of a light-harvesting chlorophyll *a*/*b*-binding protein (Lhcb1) were altered in the mutants. However, in all mutants, the FLUORESCENT IN BLUE LIGHT (FLU), glutamyl-tRNA reductase-binding protein (GBP), and Lhcb1 accumulated to WT levels (Fig. 2c).

**Figure 3:**
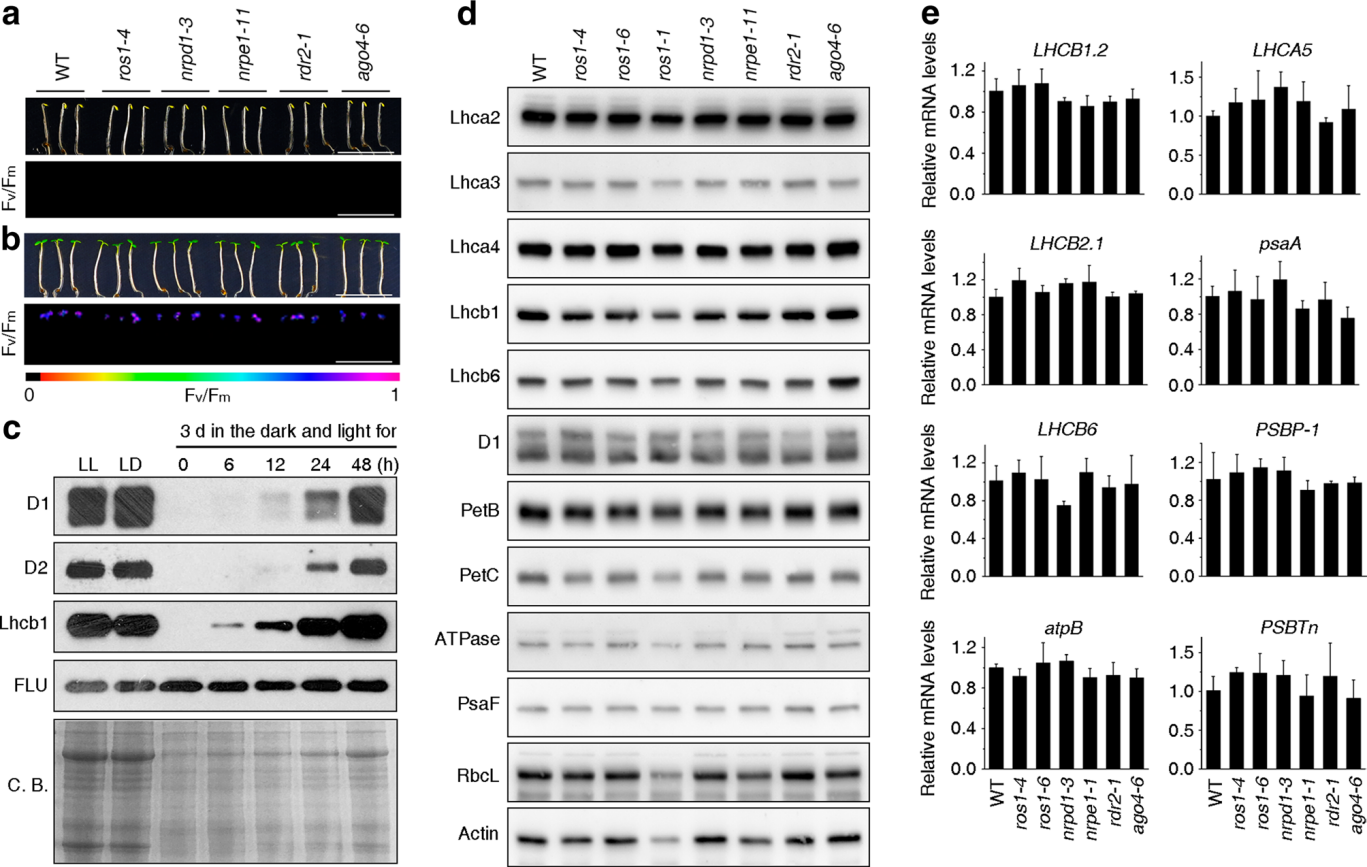
Photomorphogenesis is not significantly affected in *ros1*, *nrpd1*, *nrpe1*, *rdr2* or *ago4* mutant seedlings. **(a)** Phenotypes (upper panel) and the corresponding maximum quantum yields of PSII (F_v_/F_m_) (lower panel) of 4-day-old etiolated seedlings. F_v_/F_m_ was measured with an imaging Chl fluorometer (Imaging PAM). Scale bar = 1 cm. **(b)** Phenotypes (upper panel) and corresponding F_v_/F_m_ values (lower panel) of 3-day-old etiolated seedlings which had been exposed to continuous light for 24 h. **(c)** Immunoblot analysis of the PSII core proteins (D1 and D2), Lhcb1 and FLU during greening of etiolated seedlings. WT seedlings were grown for 3 days in the dark and exposed to light for between 0 and 48 h, as indicated. Extracted total proteins were normalized with respect to fresh weight and fractionated by SDS-PAGE. Blots were then probed with antibodies raised against the individual proteins. Total proteins from 5-day-old WT seedlings grown under continuous light (LL) and LD conditions (LD) were used as positive controls. The total protein accumulation of each sample was visualized by staining the gel with Coomassie Blue R250 (C.B.). The figure was assembled from different blots (delineated by a black rectangle) and full-length blots are presented in Supplementary Fig. S7. **(d)** Immunoblot analysis of representative photosynthesis proteins of 3-day-old etiolated mutant seedlings which had been exposed to continuous light for 24 h. Immunoblot analysis was performed as in (**c**). The figure was assembled from different blots (delineated by a black rectangle) and full-length blots are presented in Supplementary Fig. S8. **(e)** Real-time PCR analyses of 3-day-old etiolated WT (Col-0) and mutant seedlings that had been exposed to continuous light for 24 h. Real-time PCR was performed with primers specific for the nuclear genes *LHCB1.2*, *LHCB2.1*, *LHCB6*, *LHCA5*, *PSBP-1* and *PSBTn*, and the plastid genes *psaA* and *atpB*. Note that the primers for *LHCB2.1* also amplify *LHCB2.2* mRNA. Expression values are reported relative to the corresponding transcript levels in the WT and were normalized with respect to the expression level of *ACTIN2*. Data are shown as mean values ± SD from three different plant pools.

To investigate etiolated seedlings and their greening behavior, WT and *nrpd1-3*, *nrpe1-11*, *rdr2-1*, *ago4-6* and *ros1-4* mutant seedlings were grown for 3 days in darkness and then transferred into continuous light. All mutant seedlings showed the same etiolated phenotype as the WT (Fig. 3a), and were able to green to the same extent as the WT after a 24-h light treatment (Fig. 3b). To confirm these results on the molecular level, Western blot and real-time PCR analyses were performed to determine levels of representative proteins and mRNAs during the greening process. Total proteins were isolated from 3-day-old etiolated WT seedlings that had been exposed to continuous light for 0, 6, 12, 24 and 48 h, and the samples were subjected to immunoblot analyses with antibodies specific for the D1 and D2 reaction-center proteins of PSII. D1 and D2 were first detected after 12 h of light treatment, were readily detectable after 24 h, and after 48 h they had reached levels comparable to those in 5-day-old seedlings grown under LD conditions (Fig. 3c). A similar pattern was also observed for the light harvesting chlorophyll *a*/*b* binding protein Lhcb1, but FLU protein levels remained constant during the greening process (Fig. 3c). The 24-h time-point was used to quantify protein levels of PSI and PSII light harvesting complex proteins Lhca2, Lhca3, Lhca4, Lhcb1 and Lhcb6, and moreover, of representative subunits of PSI (PsaF), PSII (D1), the cytochrome *b*_6f_ complex (PetB and PetC), the chloroplast ATP synthase (γ subunit), and the large subunit of Rubisco (RbcL) in *nrpd1-3*, *nrpe1-11*, *rdr2-1*, *ago4-6* and *ros1* mutant seedlings. No difference in protein accumulation could be detected in the mutant seedlings compared to the WT (Fig. 3d). Under these conditions, real-time PCR analyses also failed to reveal any significant differences in levels of accumulation of the nucleus-encoded *LIGHT HARVESTING CHLOROPHYLL A/B BINDING PROTEINs LHCB1.2, LHCB2.1, LHCB6*, *LHCA5,* or *PSII SUBUNIT P-1* (*PSBP-1*) and T (*PSBTn*) mRNAs, or transcripts of the plastid-encoded *psaA* gene encoding one protein of the reaction center of PSI and the *atpB* gene encoding the β subunit of the ATP synthase (Fig. 3e).

Altogether, it can be concluded that inactivation of components of the canonical RdDM pathway does not obviously affect chloroplast development. This is compatible with the finding that chromatin condensation during photomorphogenesis does not detectably rely on DNA methylation-based processes^38^.

### Perturbations in the RdDM pathway do not affect *gun* signaling

**Figure 4:**
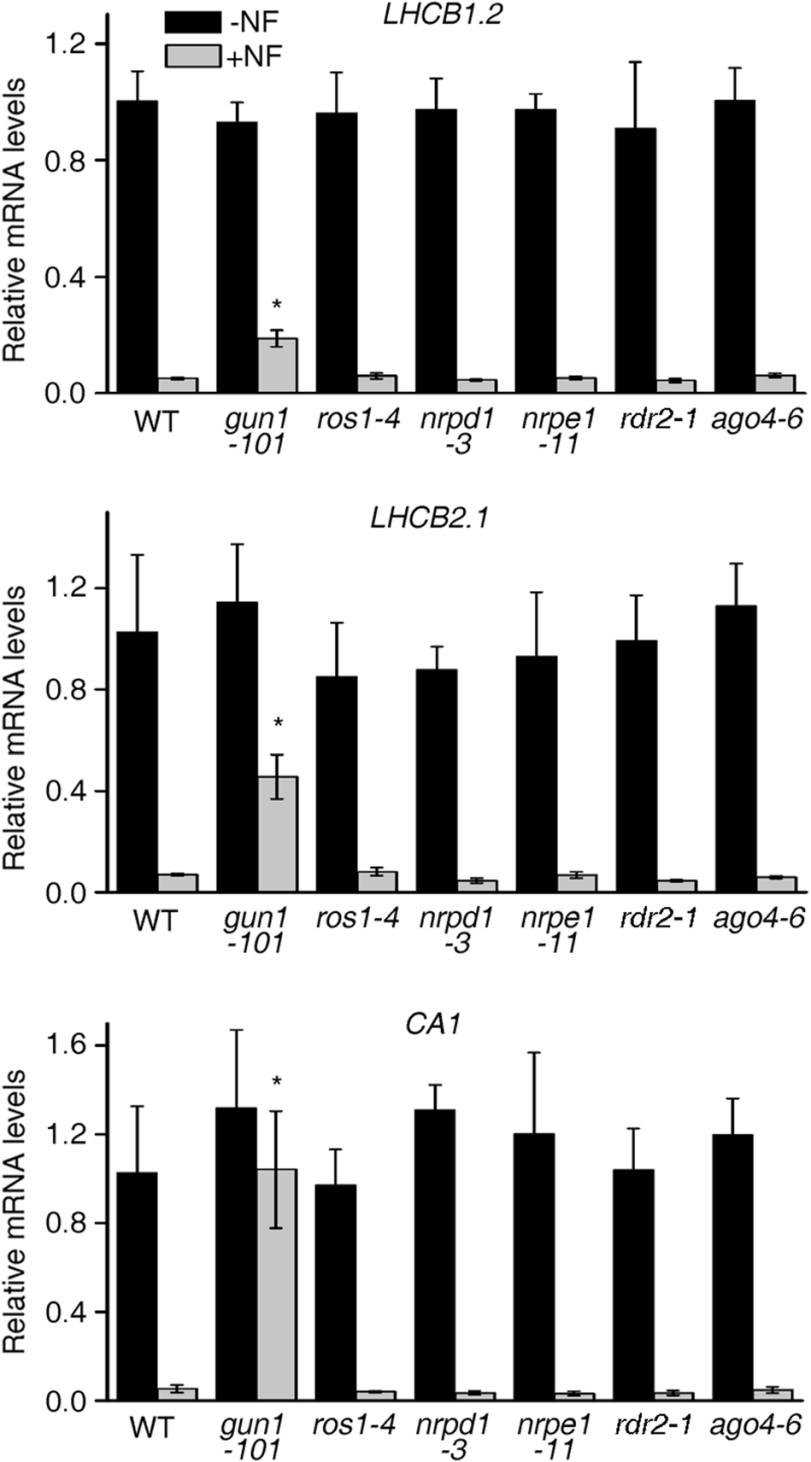
Disturbances in the RdDM pathway do not contribute to a *gun* phenotype induced by norflurazon treatment. Real-time PCR analyses of *LHCB1.2*, *LHCB2.1* and *CA1* expression in WT, *gun1-101*, *ros1-4*, *nrpd1-3*, *nrpe1-11*, *rdr2-1* and *ago4-6* seedlings grown for 6 days under LD conditions in the absence (− NF, Norflurazon) or presence of inhibitor (+ NF). Expression values are reported relative to the corresponding transcript levels in the WT grown in the absence of inhibitor and were normalized with respect to the expression level of *ACTIN2*. Data are shown as mean values ± SD from three different plant pools, each with three technical replicates. Significant differences (*t*-test*; p* < 0.05) with respect to the WT are indicated by asterisks.

Application of inhibitors like lincomycin (LIN; blocks organellar gene expression) or norflurazon (NF; blocks carotenoid biosynthesis) prevents greening of seedlings, and represses photosynthesis-related genes in the nucleus, including *LHCB1.2*, at the transcriptional level. In *genomes uncoupled* (*gun*) mutants, *LHCB1.2* expression is maintained in the presence of NF^22^. The *gun1* mutant also shows this feature under LIN treatment^39^. How *gun* mutants maintain higher *LHCB1.2* expression levels in the presence of these inhibitors is still enigmatic. It was previously shown that DNA methylation levels in genic regions affect gene expression, whereas higher promoter methylation was associated with lower levels of expression^40^. Here, we wanted to test if increased methylation of the *LHCB1.2* promoter might account for the repression of *LHCB1.2* mRNA levels after inhibitor treatment. Under this assumption, a higher degree of methylation would be expected after NF and LIN treatment of Col-0 seedlings, which should in turn be repressed in *gun1* seedlings. To test for a possible link between *LHCB1.2* promoter methylation and *LHCB1.2* mRNA levels, WT and *gun1-101* and *ago4-6* seedlings were grown for 6 days under LD conditions in the absence or presence of inhibitors. The effects of the inhibitor treatments on steady-state levels of *LHCB1.2* mRNA level were confirmed by real-time PCR analysis (see Supplementary Fig. 4a). Moreover, after treatment with LIN or NF, the *gun1* control showed enhanced *LHCB1.2* mRNA expression relative to WT plants. *LHCB1.2* mRNA levels were suppressed to the same extent as in the WT in the *ago4* mutant (see Supplementary Fig. 4a). To examine the influence of RdDM in more depth, we determined mRNA expression levels of two more target genes of *gun* signaling (*LHCB2.1* and *CARBONIC ANHYDRASE1* (*CA1*)) in addition to *LHCB1.2* in WT, *nrpd1-3*, *nrpe1-11*, *rdr2-1*, *ago4-6* and *ros1-4* seedlings grown in the absence and presence of Norflurazon (Fig. 4). While *gun1-101* showed the typical de-repressed expression of all three marker genes after inhibitor treatment compared to WT, all other mutant seedlings behaved like WT (Fig. 4).

**Figure 5:**
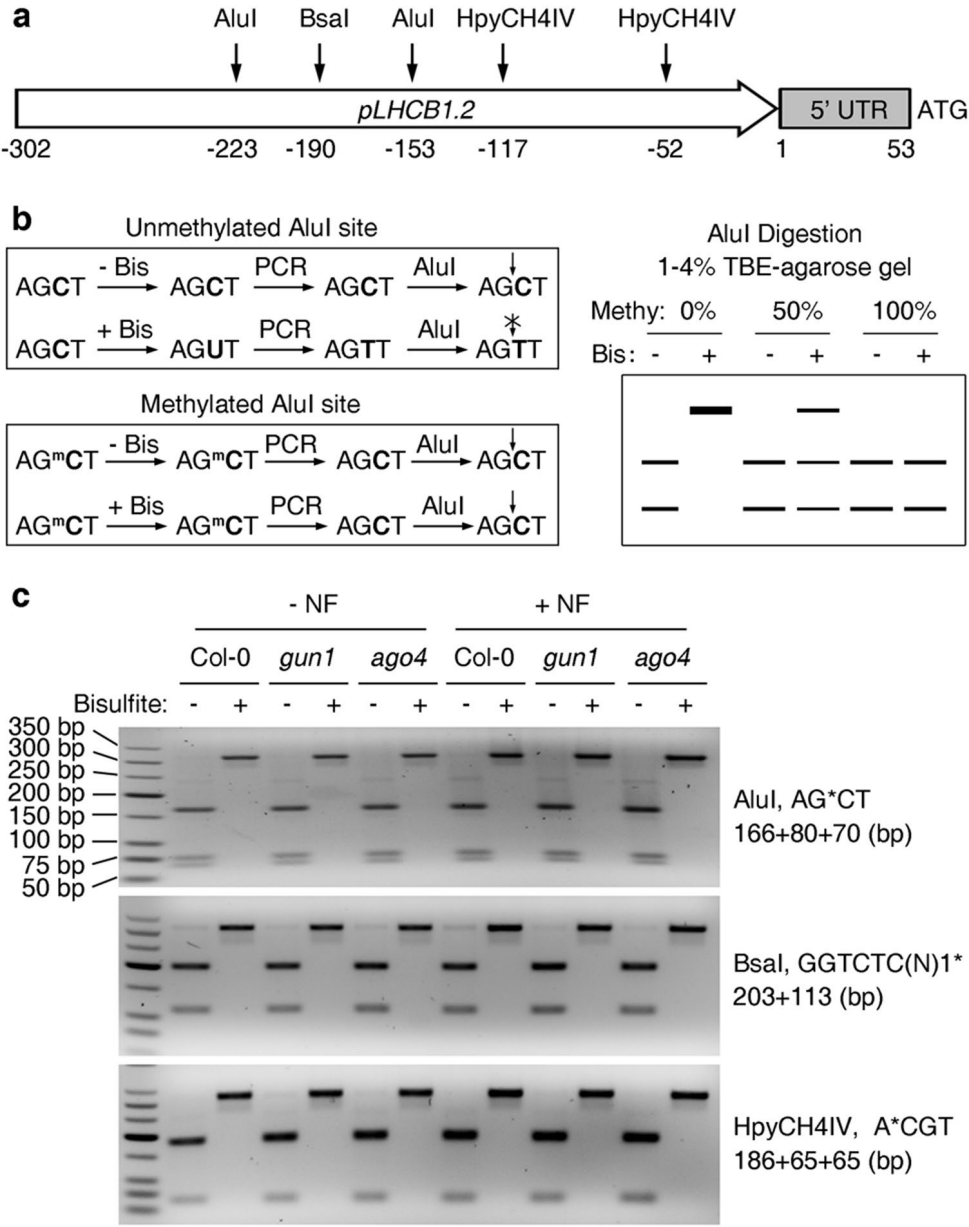
Determination of the methylation status of the core region of the *LHCB1.2* promoter. **(a)** Scheme depicting the cutting sites of the selected restriction enzymes on the *LHCB1.2* promoter. **(b)** Illustration showing the principal and work flow of COBRA (combined bisulfite restriction analysis). COBRA consists of a standard bisulfite PCR treatment which is followed by restriction enzyme digestion and quantification steps. Note that the methylation information is lost after PCR amplification when the original genomic DNA was not treated with bisulfite (Bis). The methylation status of the tested genomic DNA is transformed into visible information by running the products on an agarose gel. The AluI cutting site AGCT is used as an example. **(c)** Application of NF does not alter the methylation status of the *LHCB1.2* promoter. Seedlings of Col-0, *gun1-101* and *ago4-6* were grown on half-strength MS medium or MS medium supplied with 5 μM NF for 6 days under standard growth conditions before genomic DNA was extracted and analyzed by COBRA. Restriction enzyme cutting sites and the corresponding product sizes of the digested *LHCB1.2* promoter fragment are indicated on the right.

Moreover, determination of DNA methylation levels in the core region of the *LHCB1.2* promoter which contains the CUF1 element (CACGTA) needed for the reception of retrograde signals ^41^ showed that *LHCB1.2* promoter methylation is negligible both before and after inhibitor treatment (Fig. 5). This result was confirmed by Chop-PCR (methylation-sensitive enzyme digestion followed by PCR) in two different regions (− 52 bp and − 153 bp) of the *LHCB1.2* promoter (see Supplementary Fig. 4b, c). Therefore no correlation between *LHCB1.2* transcript accumulation and promoter methylation levels could be established. In sum, these results imply that promoter methylation levels do not affect *gun* signaling or *LHCB1.2* mRNA expression and that *gun* signaling is independent of the RdDM pathway.

## Discussion

Despite the critical significance of chloroplast biogenesis for plant development and performance, our understanding of how functional chloroplasts are initially established remains incomplete, and new layers of control are still being considered. One such proposal concerns the potential relationship between DNA methylation changes, chloroplast development and the expression of nucleus-encoded photosynthesis genes. With the advent of genome-wide studies, this issue can be addressed experimentally. Thus, genome-wide profiling of four histone modifications has uncovered a correlation between the activation of photosynthetic genes in response to light during deetiolation in Arabidopsis and increases in histone acetylation^42^. In an RNA-Seq study, genes for histone H3-K9 methylation and photosystem assembly exhibited correlated changes in particular during the final transition to photosynthetically functional chloroplasts^28^. It was suggested before that variation in genomic methylation in natural populations of *Populus simonii* is associated with leaf shape and photosynthetic traits^43^, and that histone methylation could play a role in the regulation of photosynthesis genes during needle development of *Pinus radiata*^44^. Three decades ago, the reduced expression of nucleus-encoded photosynthesis genes observed in white (relative to green) cultured cells of *Acer pseudoplatanus* was attributed to a higher degree of methylation of these genes in the former^45^. Moreover, the potential roles of DNA methylation in plant responses to a wide range of abiotic environmental stress conditions, including heat, cold, drought, high salinity, hyperosmotic stress, ultraviolet radiation stress and soil nutrient deficiency, have been investigated in a variety of plants, including Arabidopsis, maize, rice, winter wheat, *Brassica rapa*, *Brassica napus*, barley, *Populus trichocarpa* and *Quercus lobata*^16^. Accordingly, it has become clear that many stress responses seem to involve epigenetic components^46^. However, although DNA methylation is one of the best-studied types of chromatin modification, to our knowledge a possible link between DNA methylation changes and chloroplast development has not been studied in Arabidopsis prior to this work.

In this study we have used a plethora of mutants in the RdDM pathway and other (de)methylation pathways. By using the maximum quantum yield of PSII (*F*_*v*_/*F*_*m*_) as a proxy for chloroplast development, we show here that chloroplast development is not disturbed in either cotyledons or young emerging leaves of any of the mutants investigated (see Fig. 1, Supplementary Fig. S1). In addition, greening is not affected in LD-grown or etiolated seedlings exposed to light for 24 h (see Figs. 2 and 3). This is compatible with the finding that chromatin condensation during photomorphogenesis does not detectably rely on DNA methylation-based processes^38^.

It has become evident that changes in DNA methylation are associated with transcriptional regulation of genes involved in plant stress responses^16^. Indeed, it was suggested that the salt-induced transcription factor AtMYB74 in Arabidopsis is transcriptionally regulated by the RdDM pathway in response to salt stress^47^. Therefore, we investigated in the second part of our study whether perturbations of chloroplast development that repress the transcription of *LHCB1.2*, the classical marker gene for retrograde signaling research, might evoke alterations in the methylation state of its promoter. Here we found no correlation between promoter methylation levels or *gun* signaling and *LHCB1.2* mRNA expression.

It is conceivable that DNA methylation changes have a detectable impact on chloroplast development or function only under stress conditions, as has been demonstrated in the *ros1* mutant, for example. In the absence of ABA, *ros1* mutants did not exhibit any obvious developmental defects, but early seedling establishment and root growth of *ros1* mutants were hypersensitive to abscisic acid^48^. Moreover, it should be noted that the extent of DNA methylation varies extensively in all three DNA methylation contexts between different plant species^49^. For example, some Brassicaceae species have reduced CG and CHG methylation, while even CHH methylation is depleted in some Poaceae species^50^. With 30.5%, 10.0%, 3.9% methylation in CG, CHG, CHH sites, respectively, methylation ratios are relatively low in Arabidopsis compared with rice (in which 58.4% of CG, 31.0% of CHG, and 5.1% of CHH sites are methylated) and *Beta vulgaris* (beet), which displays the highest levels of DNA methylation yet measured in plants: (92.6%, 81.2%, and 18.9% in CG, CHG and CHH, respectively)^50^. Additionally, mRNA levels of several genes involved in epigenetic processes accumulate differentially throughout chloroplast development (see Fig. 1a, see Supplementary Fig. S1, Supplementary Table S1 online) and some of them are even constantly down-regulated. The mRNA expression differences might reflect the action of the corresponding proteins in distinct epigenetics processes. Moreover, regulation at different steps of gene expression, for example differential splicing or post-translational modifications like seen for Arabidopsis DCL3 and -4^51^ or human DNA methyltransferases^52^ should be considered. In sum, although we found that chloroplast development appears to proceed independently of DNA methylation changes in Arabidopsis, we cannot exclude an influence of DNA methylation levels on chloroplast development in other plant species or that overexpression of genes involved in epigenetic processes would influence chloroplast development.

## Methods

### Plant material and growth conditions

All seeds used in this study are in the Columbia (Col-0) background. T-DNA insertion mutants were obtained from The European Arabidopsis Stock Centre (https://arabidopsis.info/; see Supplementary Fig. S5, see Supplementary Table S2 online). Homozygous lines were identified using gene-specific primers and T-DNA border primers designed by using the website https://signal.salk.edu/tdnaprimers.2.html. For surface sterilization, seeds were immersed for 10 min in 0.6% (v/v) sodium hypochlorite solution containing 0.01% (v/v) Triton X-100 with gentle agitation, and were then washed four times with ddH_2_O. Then seeds were cold-treated at 4 °C for 3 days, and grown on plates containing half-strength MS agar (with 0.5% (w/v) sucrose, 0.8% (w/v) plant agar) for 3–6 days under long-day conditions (LD; 16 h light (100 μmol photons m^−2^ s^−1^)/8 h dark) at 22 °C. Etiolated seedlings were obtained by growing cold-treated seeds on half-strength MS agar plates in the dark at 22 °C for 3–4 days. For lincomycin (LIN) or norflurazon (NF) treatment, 220 μg/mL LIN (Sigma Aldrich) or 5 μM NF (Sigma Aldrich) was added to half-strength MS agar medium, and seeds were grown on these plates for 6 days under LD conditions at 22 °C.

### Determination of maximum PSII efficiency

The maximum quantum efficiency of PSII as indicated by F_v_/F_m_ was recorded using an automatic pulse-amplitude modulated (PAM) fluorometer (Imaging PAM, Walz) following the instructions of the manufacturer.

### Quantification of the chlorophyll content

About 100 mg of 4-day-old seedlings were harvested and their fresh weight was measured. Seedlings were frozen in liquid nitrogen and homogenized to a fine powder. Total chlorophyll was extracted using 1 mL of ice-cold 80% acetone. After incubation on ice and in the dark, the cell debris was removed by centrifugation at 12,000 × *g* for 10 min at 4 °C. The chlorophyll content was determined by measuring the absorbance at A_645_ and A_663_ and calculated using the following equations: Chlorophyll *a* (μg/g) = (12.7 × A663-2.69 × A645) × V/W; Chlorophyll *b* (μg/g) = (22.9 × A645-4.86 × A663) × V/W; Chlorophyll (*a* + *b*) (μg/g) = (8.02 × A663 + 20.20 × A645) × V/W. V = volume of the extract (mL); W = Weight of fresh leaves (g). 80% acetone was used as a reference.

### Western blot analysis

To extract proteins, 100 mg of 6-day-old seedlings were frozen in liquid nitrogen, and ground to a fine powder. After the addition of 500 μL of protein extraction buffer (20 mM HEPES (pH 7.4), 2 mM EDTA (pH 7.4), 2 mM EGTA (pH 7.4), 25 mM NaF, 1 mM Na_3_VO_4_, 50 mM glycerophosphate, 100 mM NaCl, 0.5% (v/v) Triton X-100, 10% (v/v) glycerol, 1 × SIGMAFAST Protease Inhibitor) to each, the samples were incubated on ice for 30 min. After centrifugation at 12,000 × *g* for 30 min at 4 °C, 300-μL aliquots of the clear supernatant were transferred to tubes and 3 μL of 1% bromophenol blue (w/v) was added to each. The samples were mixed well by vortexing, denatured at 95 °C for 5 min and cooled on ice for 2 min. A 10-μL aliquot of each was loaded onto a 12% SDS-PAGE gel, and subjected to electrophoresis at 100 V until the bromophenol blue band reached the bottom of the gel. Proteins were then transferred to a polyvinylidene difluoride (PVDF) membrane (Millipore, IPVH00010). Transferred proteins were visualized by reversible staining with Ponceau S solution (0.1% (w/v), 5% (v/v) acetic acid) for 5 min and rinsed using ddH_2_O. The membrane was then blocked for 1 h using 5% skim milk in 1 × TBS (50 mM Tris–HCl (pH 7.5), 150 mM NaCl), and incubated overnight at 4 °C using antisera against D1 (Agrisera, AS05 084), D2 (Agrisera, AS06 146), FLU, GBP and Lhcb1 (Agrisera, AS01 004), Lhca2 (Agrisera, AS01 006), Lhca3 (Agrisera, AS01 007), Lhca4 (Agrisera, AS01 008), Lhcb6 (Agrisera, AS01 010), PetB (Agrisera, AS03 034) PetC (Agrisera, AS08 330), AtpC (Agrisera, AS 08 312), PasF (AS06 104), RbcL (Agrisera, AS03 037) or Actin (Agrisera, AS13 2640). The membrane was washed for 4 × 10 min with 1 × TBS-T (50 mM Tris–HCl (pH 7.5), 150 mM NaCl, 0.1% Tween-20) at room temperature (RT). Then the membrane was incubated with anti-rabbit IgG-HRP (Santa Cruz Biotechnology, sc-2004) at RT for 1 h with slow agitation. After washing for 4 × 10 min with 1 × TBS-T, the membrane was developed with ECL substrate (Thermo Scientific, 32106) and fluorescence emission was recorded using a CCD camera (Peqlab, Fusion Fx7).

### Real-time PCR analysis

Total RNA was isolated from 6-day-old *Arabidopsis* seedlings using Direct-zol RNA MiniPrep Plus columns (Zymo Research) according to the manufacturer’s instructions. Reverse transcription was performed using 1-μg aliquots of total RNA following the procedure provided with the iScript cDNA synthesis kit (Bio-RAD, Cat. 1708890). Quantitative RT-PCR was performed using an iQ5 multicolor real-time detection system (Bio-RAD) and primers specific for the gene of interest (see Supplementary Table S3 online).

### Measurement of the DNA methylation ratio using Chop-PCR

The methylation status of selected DNA sites was determined using Chop-PCR essentially according to^53^ with primers listed in Supplementary Table S3 online. 6-day-old seedlings were frozen in liquid nitrogen and ground using a tissue lyser. After the addition of 500 μL 2% CTAB solution (2% CTAB, 100 mM Tris–HCl (pH 7.5), 1.4 M NaCl, 20 mM EDTA (pH 8.0)) to 100 mg of the sample, the mixture was incubated at 65 °C for 30 min. 500 µL chloroform was added, mixed and centrifuged at 12,000 × *g* for 10 min at room temperature. The (upper) aqueous phase was transferred to a new tube, mixed with an equal volume isopropanol, and subsequently incubated at − 20 °C for 30 min. The samples were centrifuged at 12,000 × *g* for 10 min at room temperature, and the resulting pellet was washed once with 1 mL 70% ethanol and dissolved in 400 μL ddH_2_O. To remove RNA, 1 μL RNaseA (10 mg/ml) was added to each sample which was then incubated at 37 °C for 1 h. After the addition of an equal volume phenol:chlorofom:isoamyl alcohol (24:1:1, pH 8.0), the sample was mixed well and centrifuged at 12,000 × *g* for 10 min at room temperature. The upper phase was transferred to a new tube, an equal volume chloroform was added and centrifuged at 12,000 × *g* for 10 min at room temperature. The upper phase was transferred to a new tube and an equal volume isopropanol was added. After incubation at − 20 °C for 30 min, DNA in each sample was pelleted by centrifugation at 12,000 × *g* for 10 min at room temperature. The resulting DNA pellet was washed once with 1 mL 75% ethanol, and the air-dried pellet was dissolved in 50 μL ddH_2_O.

The genomic DNA concentration was measured using a Nanodrop spectrophotometer, and equal amounts (0.5–1 μg) of genomic DNA were digested overnight with a methylation-sensitive enzyme in a 20-μL reaction mixture. After digestion, the DNA methylation level at the enzyme-targeted marker locus was quantified by quantitative PCR using the primers listed in Supplementary Table S2 online. For quantitative PCR, 1 μL of the digested DNA was used as template for a 20-μL reaction mixture, and the non-digested DNA was used as PCR template for input normalization.

### Combined bisulfite restriction analysis (COBRA)

The methylation status of the core region of the *LHCB1.2* promoter was semi-quantified by combined bisulfite restriction analysis (COBRA) adopted from^54^. Approximately 1 μg of genomic DNA was treated with the EpiMark Bisulfite Conversion Kit (NEB, E3318), and the *LHCB1.2* core promoter region was amplified from the bisulfite-converted DNA using the primer pair pLHCB1.2-F (− 302 bp) and pLHCB1.2-R (13 bp) (see Supplementary Table S3 online). The resulting PCR product was gel-purified and digested with restriction enzymes as indicated. Then the digested PCR product was separated on an 3% agarose gel and the DNA band was visualized by ethidium bromide staining.

## Supplementary information

Supplementary Information 1.

Supplementary Information 2.
