# Supplementary material for: Chloroplast development and genomes uncoupled signaling are independent of the RNA-directed DNA methylation pathway
Source: Sci Rep. 2020 Sep 22;10:15412. doi: 10.1038/s41598-020-71907-w (PMC7508864; doi:10.1038/s41598-020-71907-w)
Supplement: Supplementary file 1 — Supplementary Information 1. [file 41598_2020_71907_MOESM1_ESM.pdf]

Supplementary Information for

Chloroplast development and genomes uncoupled signaling are independent of the RNA-directed DNA methylation pathway

Liangsheng Wang, Dario Leister, Tatjana Kleine

For correspondence:

Tatjana Kleine

tatjana.kleine@lmu.de

This file includes:

Supplementary Figures S1 to S8

Supplementary Tables S1 to S3

Supplementary References

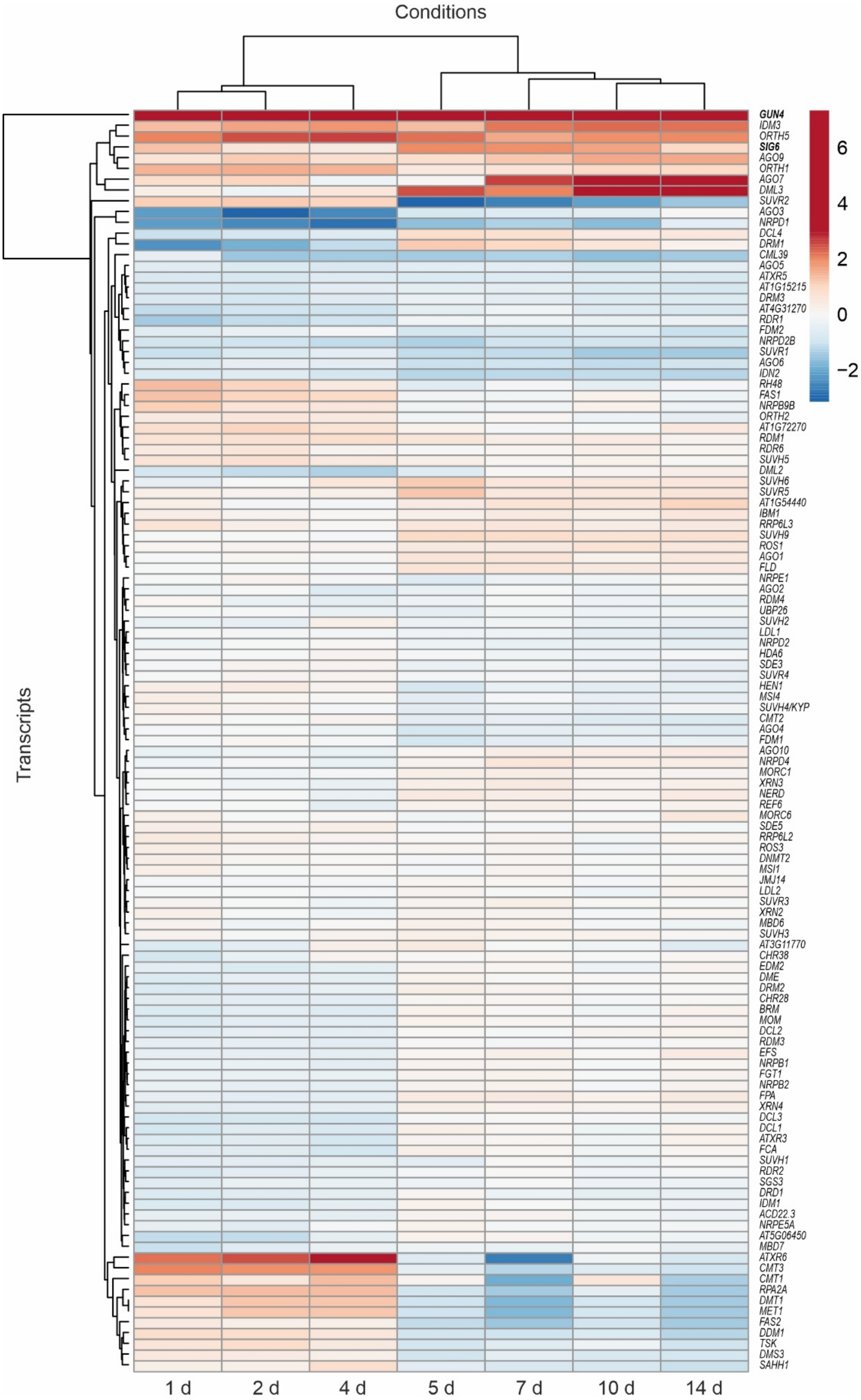

Supplementary Figure S1. Cluster analysis of relative transcript levels of genes involved in epigenetic pathways. Data for transcript levels were taken from [1]. Plotted values are log_2-fold changes compared to day 0. Clustering was performed with the help of ClustVis [2] using the Eucledian distance and the Ward method.

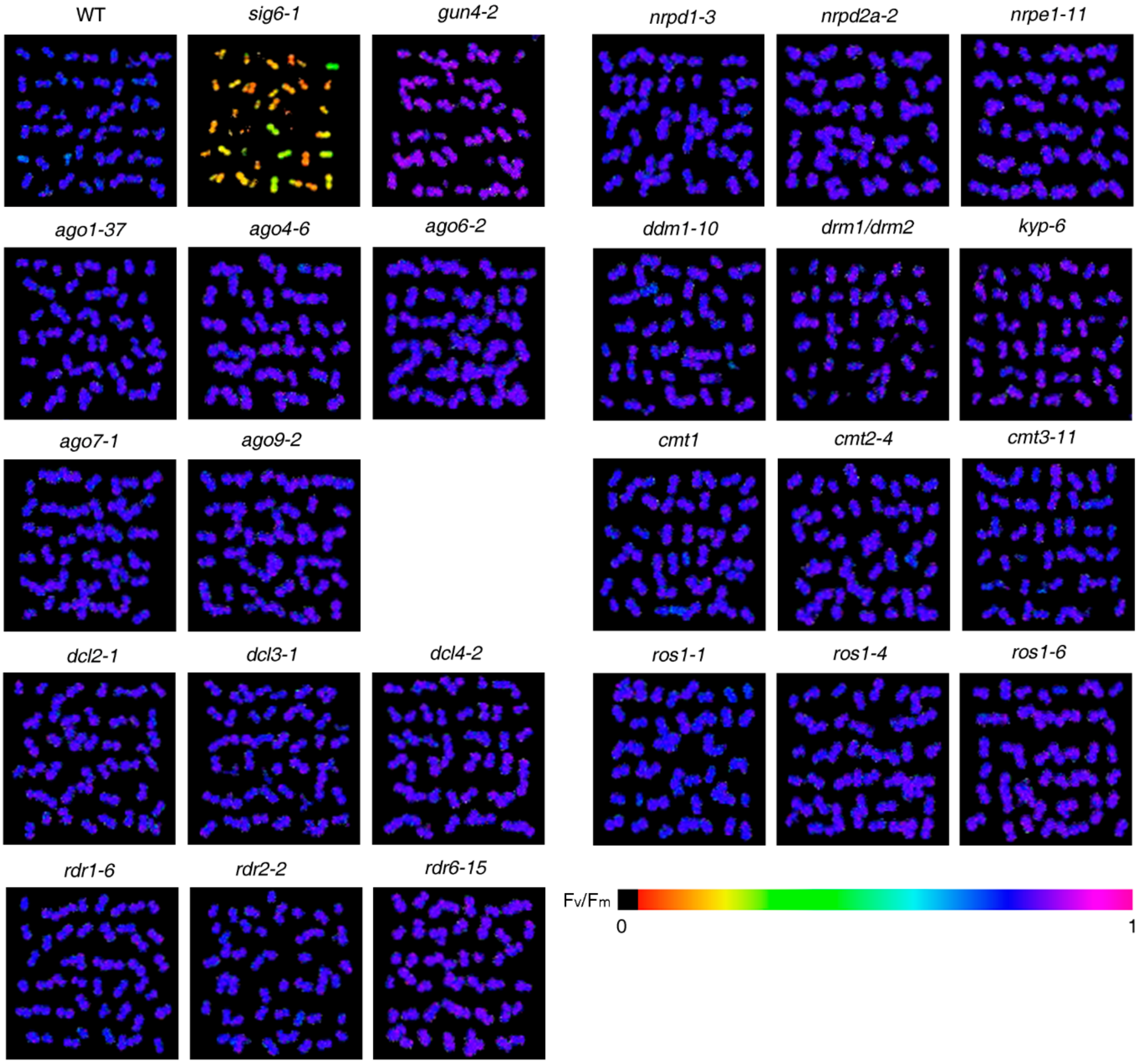

Supplementary Figure S2. Maximum quantum yield of PSII is not obviously affected in 4-day-old RdDM mutant seedlings. Imaging PAM plots showing the maximum quantum yield of PSII (F_v/F_m). Seedlings were grown under LD conditions for 4 days and dark-incubated for 10 min before chlorophyll *a* fluorescence was recorded using an automatic pulse-amplitude (PAM) fluorometer.

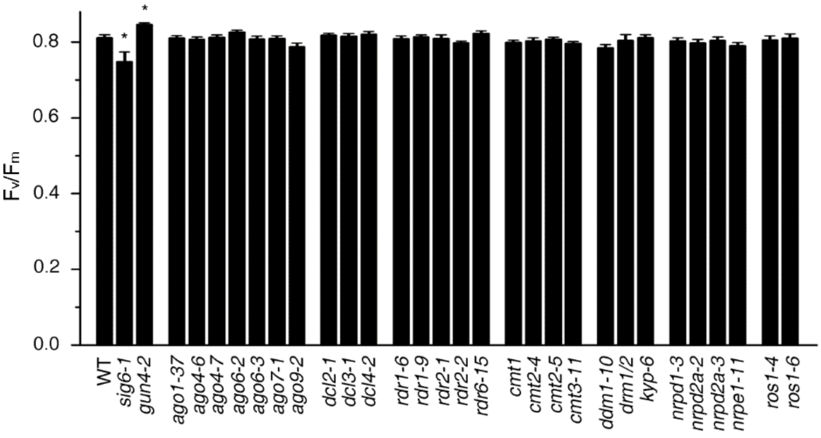

Supplementary Figure S3. Maximum quantum yield of PSII is not affected in 4-week-old RdDM mutant plants. The maximum quantum yield of PSII (F_v/F_m) was measured with an imaging Chl fluorometer (Imaging PAM). Seedlings were grown for 6 days on half-strength MS medium and were then transferred to soil and grown for another 3 weeks under LD conditions. The mature plants were dark-adapted for 10 min before transient chlorophyll fluorescence was measured. The *sig6-1* and *gun4-2* mutants were used as controls. Data represent mean values from at least 20 mature leaves. Significant differences (*t*-test; $p < 0.05$) with respect to the WT are indicated by asterisks.

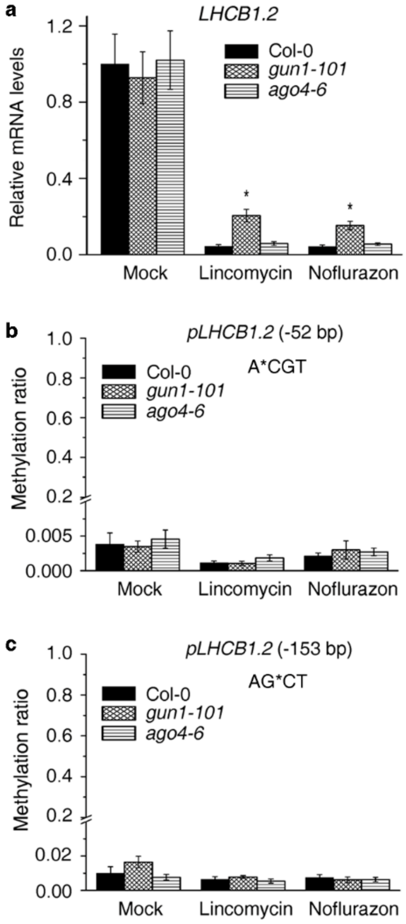

Supplementary Figure S4. Differential methylation of the *LHCB1.2* promoter does not contribute to the *gun* phenotype induced by lincomycin or norflurazon treatment.

(**a**) Real-time PCR analysis of *LHCB1.2* expression in WT, *gun1* and *ago4* seedlings grown for 6 days under LD conditions in the absence (Mock) or presence of inhibitor (Lincomycin or Norflurazon). The level of *LHCB1.2* mRNA is expressed relative to that in the WT control (grown in the absence of inhibitor) which was set to 1. The results were normalized to the expression level of *ACTIN2*. Mean values were derived from three independent experiments, each with three technical replicates. Bars indicate standard deviations. Significant differences (*t*-test*;* $p < 0.05$) with respect to the WT are indicated by asterisks.

(b, c) Determination of the methylation status of the -52 bp **(b)** and -153 bp (**c**) regions of the *LHCB1.2* promoter by Chop-PCR (methylation-sensitive enzyme digestion followed by PCR). The *gun1* mutant was used as a positive control for the effects of lincomycin and norflurazon treatment. The *ago4* mutant was used as a representative of the RdDM pathway. No significant differences could be detected by applying the *t*-test.

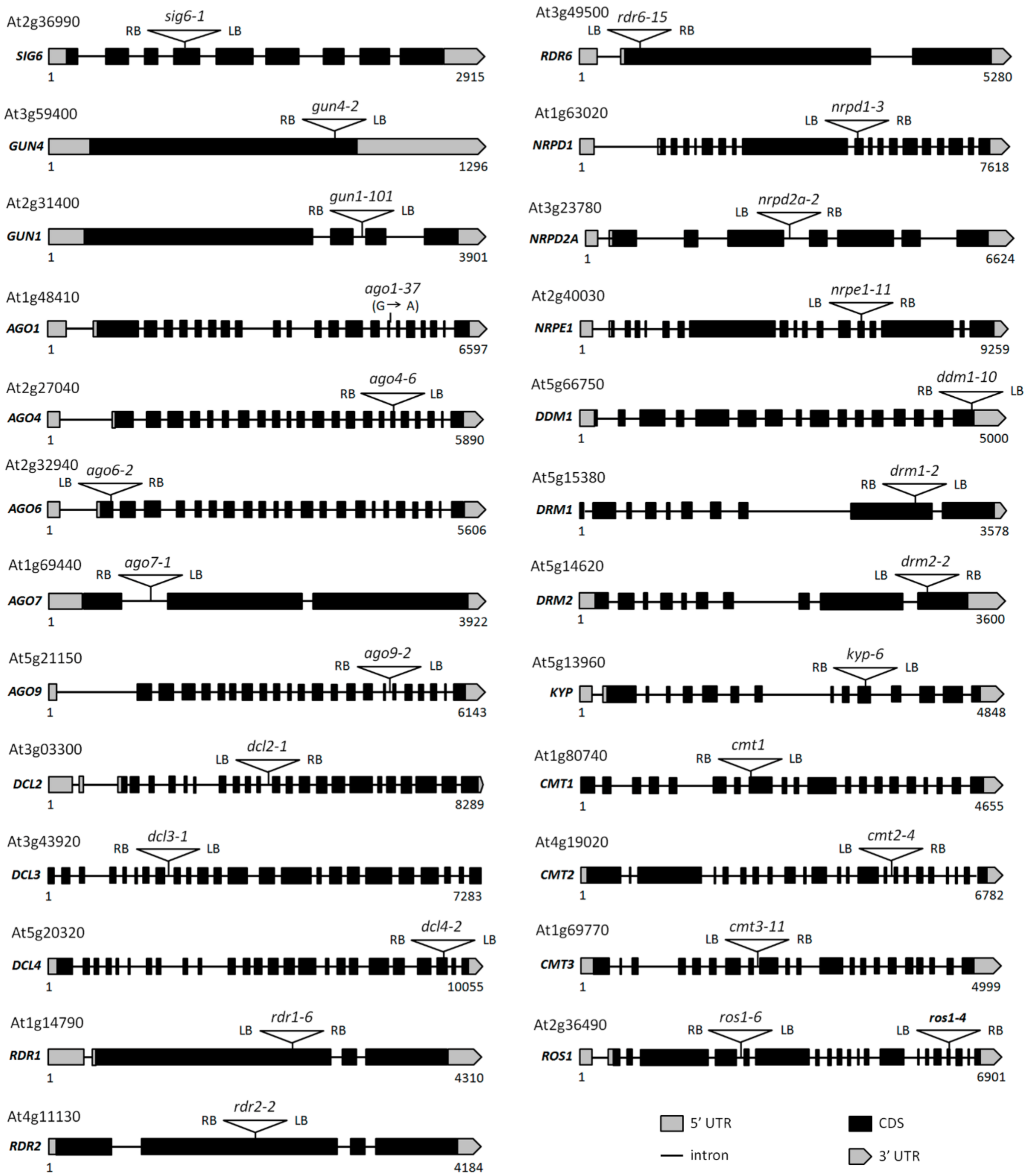

Supplementary Figure S5. Schematic representation and T-DNA tagging of the corresponding loci in the investigated mutants. Exons (black boxes), introns (black lines) and the 5' and 3' UTRs (grey boxes) are shown. Numbers are given relative to the start of the 5'UTR.

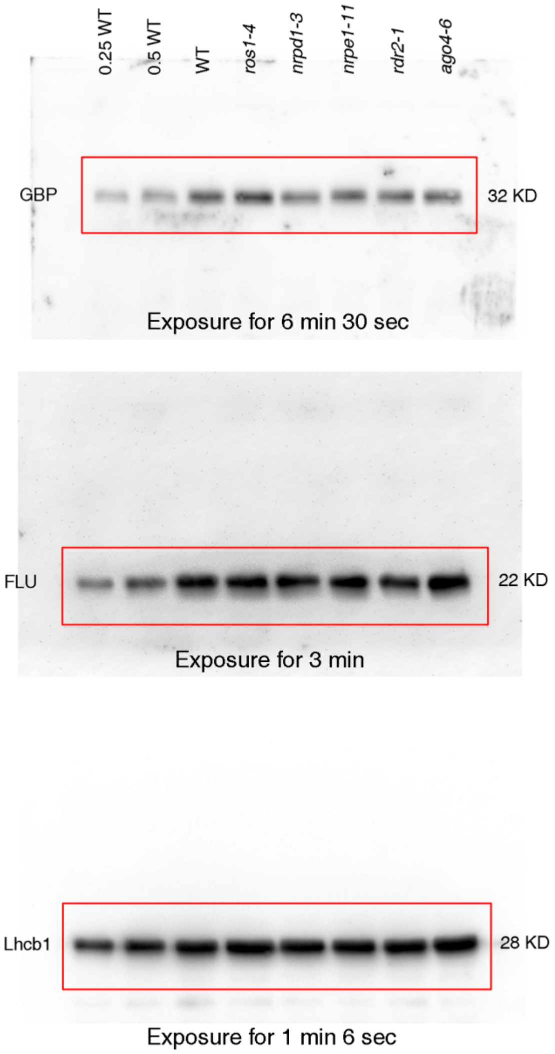

Supplementary Figure S6. Originals of Figure 2c. Cropped areas are marked by red rectangles.

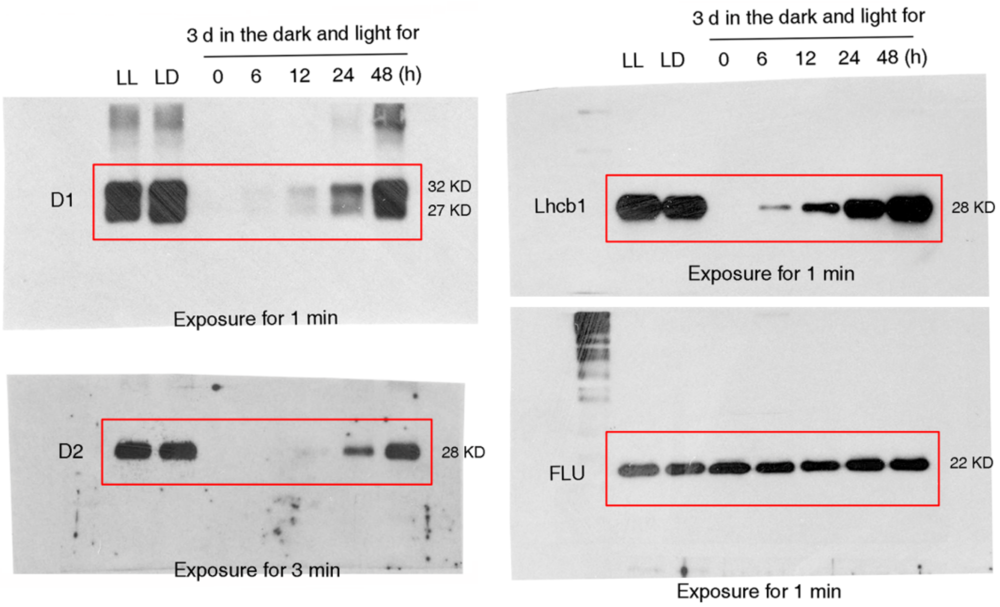

Supplementary Figure S7. Originals of Figure 3c. Cropped areas are marked by red rectangles.

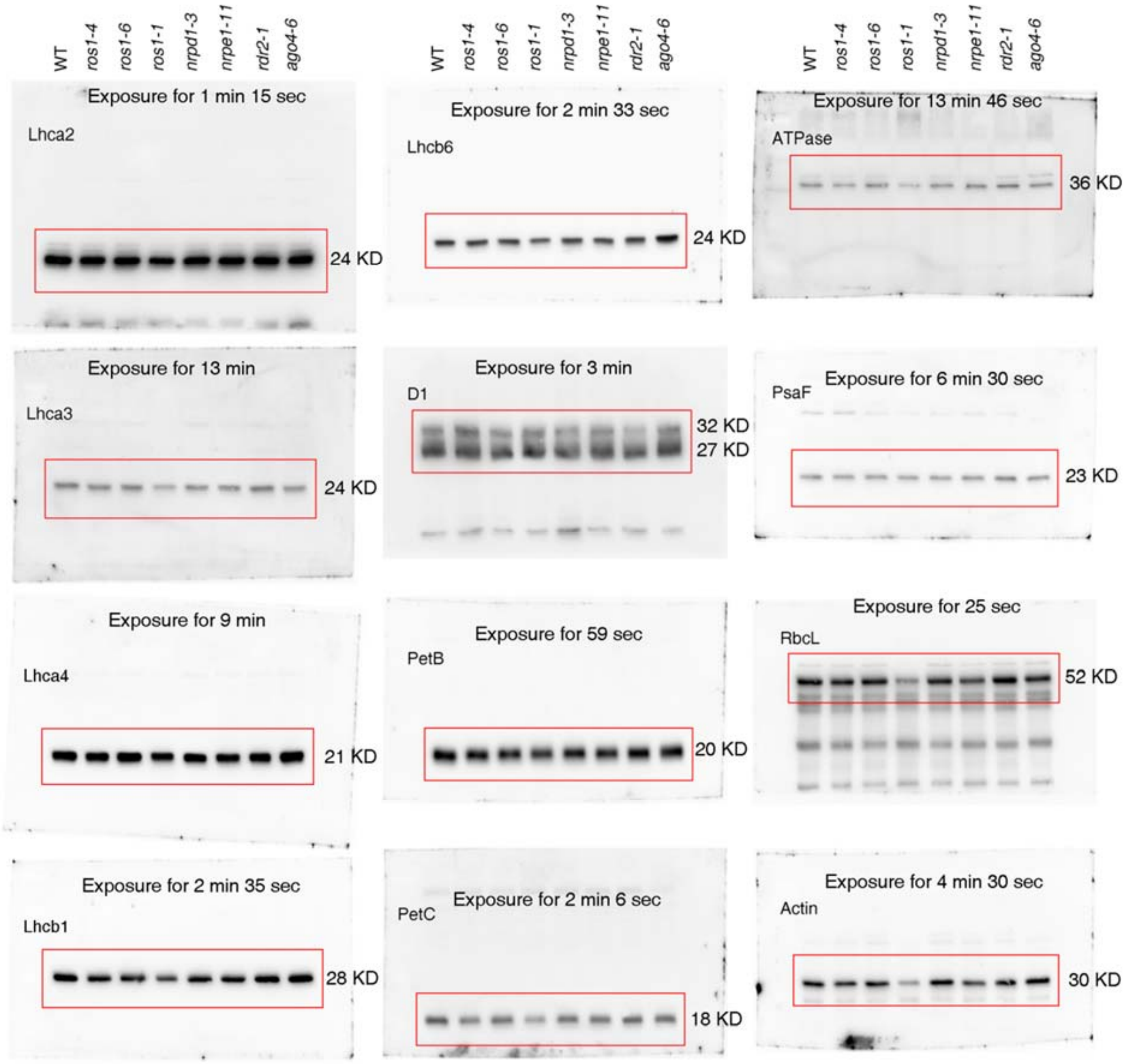

Supplementary Figure S8. Originals of Figure 3d. Cropped areas are marked by red rectangles.

Supplementary Table S1. Messenger RNA levels of genes involved in epigenetic processes. See extra Excel file.

Supplementary Table S2. Mutants used in this study.

ATG number	Stock line	Allele	Involved in	Ref.
AT2G36990	SAIL_893_C09	*sig6-1*	Early chloroplast development	[3]
AT3G59400	SALK_011461	*gun4-2*	Tetrapyrrole biosynthesis, plastid retrograde signaling	[4]
AT2G31400	SAIL_33_D01	*gun1-101*	Plastid retrograde signaling	[5,6]
AT1G48410	CS16278	*ago1-37*	Gene silencing, lateral organ development	[7]
AT2G27040	GK-066H12	*ago4-6*	RdDM, siRNA binding, defense responses	[8]
AT2G32940	SALK_031533	*ago6-2*	RdDM, siRNA binding, defense responses	[8]
AT1G69440	SALK_037458	*ago7-1*	RNA interference, miRNA and ta-siRNA generation	[9]
AT5G21150	SALK_112059	*ago9-2*	siRNA binding, defense response, female gamete formation	[10]
AT3G03300	SALK_064627	*dcl2-1*	RdDM, siRNA generation	[11]
AT3G43920	SALK_005512	*dcl3-1*	RdDM, siRNA generation	[11]
AT5G20320	GK-160G05	*dcl4-2*	RdDM, siRNA generation	[11]
AT1G14790	SALK_112300	*rdr1-6*	RNA interference, siRNA generation, defense responses	[12]
AT4G11130	SALK_059661	*rdr2-2*	RdDM, siRNA generation	[13]
AT3G49500	SAIL_617_H07	*rdr6-15*	RdDM, siRNA generation	[13]
AT1G63020	SALK_128428	*nrpd1-3*	RdDM, siRNA generation	[14]
AT3G23780	SALK_046208	*nrpd2a-2*	RdDM, siRNA generation	[15]
AT2G40030	SALK_029919	*nrpe1-11*	RdDM, particularly CHH (H=A, T or C) methylation	[16]
AT5G66750	SALK_093009	*ddm1-10*	Gene silencing, DNA methylation, histone methylation	[17]
AT5G15380/ AT5G14620	SALK_031705/ SALK_150863	*drm1-2/ drm2-2*	De novo DNA methylation in CG, CHG and CHH context, gene silencing	[18]
AT5G13960	SALK_041474	*kyp-6*	Maintenance of DNA methylation (CG, CHG and CHH)	[19]
AT1G80740	SALK_030404	*cmt1*	DNA methylation, DNA mediated-transformation	[20]
AT4G19020	SALK_201637	*cmt2-4*	Methylation of cytosines in CHH contexts, heat tolerance	[21]
AT1G69770	SALK_148381	*cmt3-11*	Methylation of cytosines at non-CG sites	[22]
AT2G36490	SALK_045303	*ros1-4*	DNA demethylation	[23]
AT2G36490	WiscDsLox469C11	*ros1-6*	DNA demethylation	[23]

Supplementary Table S3. Primers used in this study.

	Forward primer (5'-3')	Reverse primer (5'-3')
**Real-time PCR**		
LHCB1.2	GACTTTCAGCTGATCCCGAG	CGGTCCCTTACCAGTGACAA
LHCB2.1/LHCB2.2	CCAAAGCATCTGGTATGGAC	CGGCTTCTCCGAATTTAACAC
LHCB6	GCATGGTTTGAAGCTGGAGC	ACAAACCAAGAGCACCGAGA
LHCA5	GCAAAATGGCCGTAGTTTTACGTGG	GCCACGTGGCACGTTCAACAG
PSBP1	ATGGAGATGGGTTCAAAGTG	TAGGAGGTAATTAACCTGAGAG
PSBT2	CCCAGCTTAGAAGTCAAGGA	ACTTGTGCGTATTTCTTCTTGG
CA1	GAGAAATACGAAACCAACCCT	ACATAAGCCCTTTGATCCCA
atpB	TATCGCCCAAATCATTGGTC	ACTCATAGCTACAGCTCTAACTC
psaA	AACCAATTTCTAAACGCTGG	TGATGATGTGCTATATCGGT
Actin2	TGTGTGACAAACTCTCTGGG	GGCATCAATTCGATCACTCAG
**Chop-PCR**		
pLHCB1.2 (-52 bp)	TCCAATGAGTAAAGAGATATAGATTACTTCATAG	CTTCCGAGGACTTCAGATGCCG
pLHCB1.2 (-153 bp)	GGTCTCGAAATGCTTTGGCTGC	CTATGAAGTAATCTATATCTCTTTACTCATTGGA
**COBRA**		
pLHCB1.2-F (-302-to-13 bp)	TCATCAATATCACACTATCTTTACTTTTATTTAA	TTGTGAGTGAGAGTGATTGAAAATGGTT

Supplementary References

1 Dubreuil, C. *et al.* Establishment of Photosynthesis through Chloroplast Development Is Controlled by Two Distinct Regulatory Phases. *Plant Physiol* **176**, 1199-1214, doi:10.1104/pp.17.00435 (2018).

2 Metsalu, T. & Vilo, J. ClustVis: a web tool for visualizing clustering of multivariate data using Principal Component Analysis and heatmap. *Nucleic Acids Res* **43**, W566-570, doi:10.1093/nar/gkv468 (2015).

3 Ishizaki, Y. *et al.* A nuclear-encoded sigma factor, Arabidopsis SIG6, recognizes sigma-70 type chloroplast promoters and regulates early chloroplast development in cotyledons. *Plant J* **42**, 133-144, doi:10.1111/j.1365-313X.2005.02362.x (2005).

4 Peter, E. & Grimm, B. GUN4 is required for posttranslational control of plant tetrapyrrole biosynthesis. *Mol Plant* **2**, 1198-1210, doi:10.1093/mp/ssp072 (2009).

5 Shimizu, T. *et al.* The retrograde signaling protein GUN1 regulates tetrapyrrole biosynthesis. *P Natl Acad Sci USA* **116**, 24900-24906, doi:10.1073/pnas.1911251116 (2019).

6 Ruckle, M. E., DeMarco, S. M. & Larkin, R. M. Plastid signals remodel light signaling networks and are essential for efficient chloroplast biogenesis in Arabidopsis. *Plant Cell* **19**, 3944-3960, doi:10.1105/tpc.107.054312 (2007).

7 Li, Y. *et al.* Characterizations of a hypomorphic argonaute1 mutant reveal novel AGO1 functions in Arabidopsis lateral organ development. *Plant Molecular Biology* **61**, 63-78, doi:10.1007/s11103-005-5992-7 (2006).

8 Duan, C. G. *et al.* Specific but interdependent functions for Arabidopsis AGO4 and AGO6 in RNA-directed DNA methylation. *Embo Journal* **34**, 581-592, doi:10.15252/embj.201489453 (2015).

9 Jouannet, V. *et al.* Cytoplasmic Arabidopsis AGO7 accumulates in membrane-associated siRNA bodies and is required for ta-siRNA biogenesis. *Embo Journal* **31**, 1704-1713, doi:10.1038/emboj.2012.20 (2012).

10 Olmedo-Monfil, V. *et al.* Control of female gamete formation by a small RNA pathway in Arabidopsis. *Nature* **464**, 628-U200, doi:10.1038/nature08828 (2010).

11 Ye, R. Q. *et al.* A dicer-independent route for biogenesis of siRNAs that direct DNA methylation in Arabidopsis. *Mol Cell* **61**, 222-235, doi:10.1016/j.molcel.2015.11.015 (2016).

12 Leibman, D. *et al.* Differential expression of cucumber RNA-dependent RNA polymerase 1 genes during antiviral defence and resistance. *Mol Plant Pathol* **19**, 300-312, doi:10.1111/mpp.12518 (2018).

13 Willmann, M. R., Endres, M. W., Cook, R. T. & Gregory, B. D. The functions of RNA-dependent RNA polymerases in Arabidopsis. *The arabidopsis book* **9**, e0146, doi:10.1199/tab.0146 (2011).

14 Dalmay, T., Hamilton, A., Rudd, S., Angell, S. & Baulcombe, D. C. An RNA-Dependent RNA polymerase gene in Arabidopsis is required for posttranscriptional gene silencing mediated by a transgene but not by a virus. *Cell* **101**, 543-553, doi:Doi 10.1016/S0092-8674(00)80864-8 (2000).

15 Ream, T. S. *et al.* Subunit compositions of the RNA-silencing enzymes Pol IV and Pol V reveal their origins as specialized forms of RNA polymerase II. *Mol Cell* **33**, 192-203, doi:10.1016/j.molcel.2008.12.015 (2009).

16 Sasaki, E., Kawakatsu, T., Ecker, J. R. & Nordborg, M. Common alleles of CMT2 and NRPE1 are major determinants of CHH methylation variation in Arabidopsis thaliana. *Plos Genet* **15**, doi:ARTN e100849210.1371/journal.pgen.1008492 (2019).

17 Lyons, D. B. & Zilberman, D. DDM1 and Lsh remodelers allow methylation of DNA wrapped in nucleosomes. *Elife* **6**, doi:ARTN e3067410.7554/eLife.30674 (2017).

18 Cao, X. F. & Jacobsen, S. E. Role of the Arabidopsis DRM methyltransferases in de novo DNA methylation and gene silencing. *Current Biology* **12**, 1138-1144, doi:Pii S0960-9822(02)00925-9. doi 10.1016/S0960-9822(02)00925-9 (2002).

19 Jackson, J. P., Lindroth, A. M., Cao, X. & Jacobsen, S. E. Control of CpNpG DNA methylation by the KRYPTONITE histone H3 methyltransferase. *Nature* **416**, 556-560, doi:10.1038/nature731 (2002).

20 Crane, Y. M. & Gelvin, S. B. RNAi-mediated gene silencing reveals involvement of Arabidopsis chromatin-related genes in Agrobacterium-mediated root transformation. *Proc Natl Acad Sci U S A* **104**, 15156-15161, doi:10.1073/pnas.0706986104 (2007).

21 Shen, X. *et al.* Natural CMT2 variation is associated with genome-wide methylation changes and temperature seasonality. *Plos Genet* **10**, doi:ARTN e100484210.1371/journal.pgen.1004842 (2014).

22 Bartee, L., Malagnac, F. & Bender, J. Arabidopsis cmt3 chromomethylase mutations block non-CG methylation and silencing of an endogenous gene. *Genes Dev* **15**, 1753-1758, doi:10.1101/gad.905701 (2001).

23 Gong, Z. H. *et al.* ROS1, a repressor of transcriptional gene silencing in Arabidopsis, encodes a DNA glycosylase/lyase. *Cell* **111**, 803-814, doi:Doi 10.1016/S0092-8674(02)01133-9 (2002).
